# Synthesis and Characterization of Ti-Sn Alloy for Orthopedic Application

**DOI:** 10.3390/ma14247660

**Published:** 2021-12-12

**Authors:** Ambreen Azmat, Muhammad Tufail, Ali Dad Chandio

**Affiliations:** Materials and Surface Engineering Research Lab, Department of Metallurgical Engineering, NED University of Engineering and Technology, Karachi 75270, Pakistan; azmatamber@neduet.edu.pk (A.A.); pvc@neduet.edu.pk (M.T.)

**Keywords:** powder metallurgy, XRD, Ti-Sn alloy, corrosion rate, orthopedic, intermetallic compounds

## Abstract

Titanium (Ti)-based alloys (e.g., Ti6Al4V) are widely used in orthopedic implant applications owing to their excellent mechanical properties and biocompatibility. However, their corrosion resistance needs to be optimized. In addition, the presence of aluminum and vanadium cause alzheimer and cancer, respectively. Therefore, in this study, titanium-based alloys were developed via powder metallurgy route. In these alloys, the Al and V were replaced with tin (Sn) which was the main aim of this study. Four sets of samples were prepared by varying Sn contents, i.e., 5 to 20 wt. %. This was followed by characterization techniques including laser particle analyzer (LPA), X-ray diffractometer (XRD), scanning electron microscope (SEM), computerized potentiostate, vicker hardness tester, and nanoindenter. Results demonstrate the powder sizes between 50 and 55 µm exhibiting very good densification after sintering. The alloy contained alpha at all concentrations of Sn. However, as Sn content in the alloy exceeded from 10 wt. %, the formation of intermetallic compounds was significant. Thus, the presence of such intermetallic phases are attributed to enhanced elastic modulus. In particular, when Sn content was between 15 and 20 wt. % a drastic increase in elastic modulus was observed thereby surpassing the standard/reference alloy (Ti6Al4V). However, at 10 wt. % of Sn, the elastic modulus is more or less comparable to reference counterpart. Similarly, hardness was also increased in an ascending order upon Sn addition, i.e., 250 to 310 HV. Specifically, at 10 wt. % Sn, the hardness was observed to be 250 HV which is quite near to reference alloy, i.e., 210 HV. Moreover, tensile strength (TS) of the alloys were calculated using hardness values since it was very difficult to prepare the test coupons using powders. The TS values were in the range of 975 to 1524 MPa at all concentrations of Sn. In particular, the TS at 10 wt. % Sn is 1149 MPa which is comparable to reference counterpart (1168 MPa). The corrosion rate of Titanium-Sn alloys (as of this study) and reference alloy, i.e., Ti6Al4V were also compared. Incorporation of Sn reduced the corrosion rate at large than that of reference counterpart. In particular, the trend was in decreasing order as Sn content increased from 5 to 20 wt. %. The minimum corrosion rate of 3.65 × 10^−9^ mm/year was noticed at 20 wt. % than that of 0.03 mm/year of reference alloy. This shows the excellent corrosion resistance upon addition of Sn at all concentrations.

## 1. Introduction

Biomaterials are one of the very important classes of engineering materials owing to certain properties that make them appropriate for various applications such as dentistry and orthopedic surgeries. Such properties include biocompatibility, high strength, fatigue resistance, low elastic modulus [1], and excellent corrosion resistance [2]. Commonly employed biomaterials include gold, stainless steel (SS), and cobalt chromium (CoCr) alloys [3,4,5,6]. However, these alloys exhibit poor corrosion resistance and higher elastic moduli (i.e., SS = 205 GPa [7] and Co-Cr-Mo alloys = 230 GPa [8]) which results in revision surgery due to the stress shielding phenomenon [9]. This phenomenon takes place when the implant material transfers the major portion of the load resulting in unstressed tissue around the implant, leading to osteopenia and eventual implant failure [10].

On the contrary, titanium alloys are be promising materials for both orthopedic and dental implant applications. This is because of their excellent biocompatibility, and desired mechanical properties such as low elastic modulus and improved resistance to corrosion [11,12,13]. Currently there are several variants of titanium alloys that are available such as Cp-Ti [14], Ti6Al4V [15], Ti-Nb-Sn [16], Ti-Nb-Zr [17], Ti-Nb [18], Ti-Fe [19], Ti Mo [20], Ti-Cu [21], and Ti-Fe-Nb [22]. However, Cp-Ti and Ti6Al4V are predominantly utilized clinically owing to their enhanced mechanical properties such as high strength, low modulus of elasticity, improved biocompatibility, and excellent resistance to corrosion [23,24,25,26]. In general, titanium exists in two different crystallographic forms, i.e., below 883 °C it is HCP (alpha phase) whereas above 883 °C it changes to BCC structure (beta phase) [27,28]. Nevertheless, such polymorphs need to be stabilized by the addition of certain elements such as Nb, Ta (for beta structure), Al, O, and N (for alpha phase) [29,30,31]. Therefore, these elements are known to be either alpha or beta stabilizers.

Moreover, when aluminum and vanadium ions are present in the alloy that is being implanted into the physical body they poses certain issues [32]. This is because of their interaction with the surrounding tissues: the release of such ions is highly inflammatory. Thereafter these ions are accumulated in human body causing serious issues such as Alzheimer’s disease and cancer upon their long-term implantation [33,34,35,36,37,38]. On the contrary, several studies suggested beneficial effects of tin when added into titanium alloys [39]. For example, a study showed that tin is a neutral stabilizer and it does not create toxicity when added in the human body [40]. Production of implants of titanium alloys are usually carried out using two basic routes/techniques, i.e., powder metallurgy and casting [41]. The oldest method to fabricate implant material is casting, owing to the ability to fabricate complex shapes [42]. This involves the selection of chemical composition/elements and their melting in a vacuum furnace followed by casting/pouring into desired molds. Once the casting is completed, secondary operations can begin, such as machining. The cost of machining and other expensive thermo-mechanical processes increases the overall cost of the component [43]. In addition, it can be difficult to maintain the precise chemical composition which necessitates additional steps. Therefore, to reduce the processing cost, the powder metallurgy technique is a widely accepted method as it can produce near net parts or components. This means there is almost no need to have parts machined [44]. However, in some cases, such as when groves or tapered holes are needed, machining may be employed [45].

Thus, in this study, powder metallurgy was chosen to produce titanium tin-based alloys. This is because it is possible to have precise chemical composition using this method [46]. As noted earlier, Ti-Sn alloy was prepared using a casting route which did not yield successful results, as the casting technique introduces defects. In addition, to the knowledge of authors, there are no previous studies on Ti-Sn alloys produced via powder metallurgy where alloys are tested using simulated body fluid (SBF) mediums. However, several studies used different mediums such as the ringer solution, for example, L. C. Tsao et al. published data on Ti-15Sn alloys in ringer solution with a Icorr value (current density) of 291.8 nA/cm^2^ [47].

Therefore, in this study, four variations of Sn in the range of 5%, 10%, 15% and 20% were incorporated into titanium to improve mechanical properties such as elastic modulus, tensile strength, and hardness, in addition to corrosion resistance. These characteristics are compared with commercially available grade of pure titanium and Ti6Al4V alloy.

## 2. Materials and Methods

### 2.1. Materials

The titanium and tin powders were purchased from Sigma Aldrich, Burlington, MA, USA to form the alloys as discussed earlier. Titanium was chosen as the base material and tin as an alloying element. These powders were used in their as-received condition. The details of these materials/elements are placed in Table 1.

### 2.2. Methods

The alloys were developed by varying weight percentages of tin via powder metallurgy route. The details of such alloys are shown in Table 2.

Before alloying, the particle sizes of these powders were examined using laser particle analyzer (BT-9300H). Next, the powders were weighted in the vacuum glove box (TOB-STX2) in the presence of an argon environment to avoid the oxidation and contamination of powders. Subsequently, the alloys were blended in vacuum chamber (Desktop Vertical Automatic Mixer model: 4 Tank Mixer, MTI Corporation, Richmond, CA, USA) for 2 h at 200 rpm to mix them homogenously. It should be noted that zinc stearate was used as to de-agglomerate the powder particles. This was followed by consolidation of powders for different alloys as noted in Table 2 by cold isostatic pressing using the K100 die (l = 148 mm, OD = 65 mm and ID = 20 mm) to form the green compacts (20 mm diameter and 10 mm height) at a pressure of 20 MPa for 30 min. Stearic acid (1 wt. %) was also used as a lubricant during compaction since it improves the compressibility.

**Figure 1:**
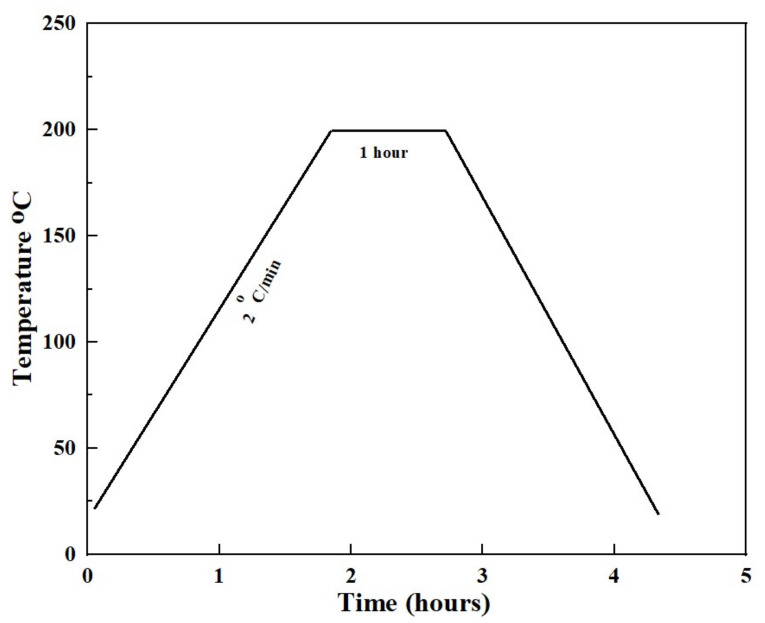
Calcination cycle of Ti-Sn alloy.

Afterwards, calcination of the green compacts were carried out to remove the binders at a temperature of 200 °C at ramp rate of 2 °C/min for an hour in an argon environment as shown in Figure 1.

**Figure 2:**
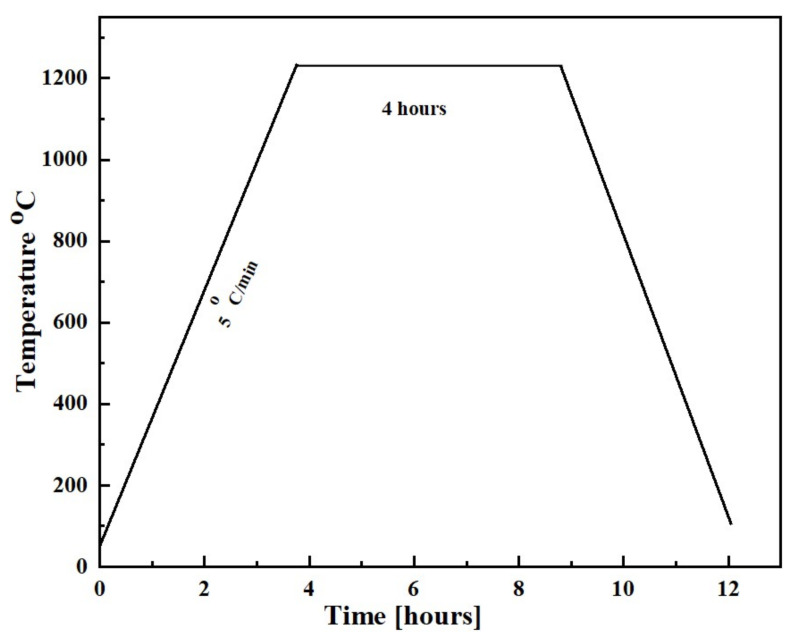
The sintering cycle of the Ti-Sn alloys.

This was followed by the sintering operations at 1200 °C with a heating rate of 5 °C/min for 4 h in the presence of an argon atmosphere in a tube furnace. The sintering cycle is shown in Figure 2. The purpose of the sintering was to develop the finished alloys or to bind together the powder particles.

### 2.3. Phase Composition Analysis

The alloys were subjected to XRD analysis (PANanalytcial X-pert Pro XRD DY3313, Amsterdam, The Netherlands,) for the determination of their phases. The diffraction patterns were recorded over 2θ at the scanning rate of 0.1°/second and in range from 10 to 80°.

### 2.4. Microstructural Analysis

Sintered samples and powders were characterized by SEM (TESCAN, Series; Vega-3, Prague, Czech Republic) to examine their morphology and other microstructural details. The samples were ground between 120 to 1000 grit SiC papers. This was followed by polishing using alumina solutions (1–0.5 µm) and etching by Kroll’s reagent (HF(2 mL), HNO_3_ (4 mL) and H_2_O 100 mL) [48] for 30 s to reveal their microstructures.

### 2.5. Relative Density

The relative density of the Ti-Sn alloys were calculated using the Equation (1) [49] and total porosity was determined using Equation (2) [50].

(1) $$\rho_{r}=\frac{\rho_{g}}{\rho_{s}}$$

where ρ_r_ = Relative density, ρ_s_ = Sintered density, ρ_g_ = Green compact density, and

(2) $$P_{t}=100\;\left(1-\frac{\rho_{s}}{\rho_{t}}\right)$$

where P_t_ = Total Porosity, $\rho_{s}$ = Green compact density, $\rho_{t}$ = theoretical density.

### 2.6. Corrosion Testing

**Figure 3:**
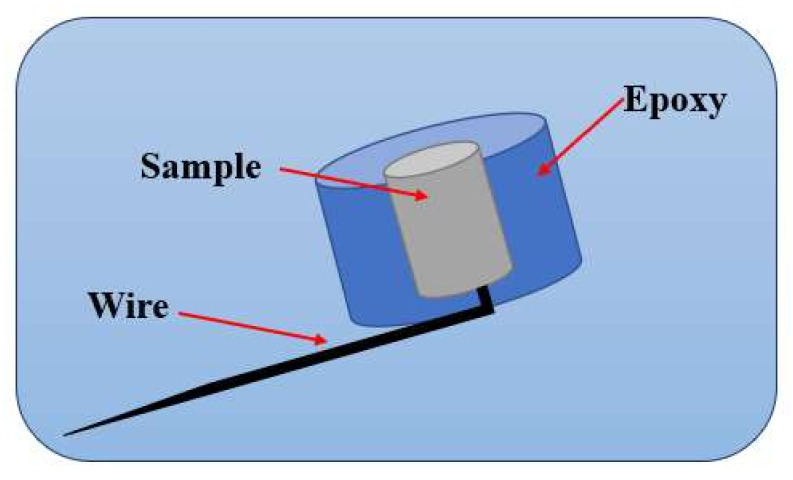
Schematic diagram of the working electrode for potentiostate.

Corrosion rate was determined via potentiostate (G 750) using the simulated body fluid (SBF) solution having 7.4 pH in the voltage range of −0.3 to 0.3 V. The composition of the SBF was prepared according to the kukuboo recipe as given in Table 3 [51]. Preparation of samples (i.e., the working electrodes) were done by following ASTM G108-94 standard [52] and analyzed at 37 °C as shown in Figure 3. Tafel curves were plotted at a rate of 1 mv/sec to find the current-density and potential. The rate of corrosion is calculated using Equation (3) [53].

(3) $$Rate\;of\;Corrosion=\frac{0.00397\times Equivalent\;weight\times Current\;density}{Density}$$

### 2.7. Elastic Modulus/Tensile Strength (TS)

Nano indentation method (TTX-NHT3) was used to find out the modulus of elasticity (E) of samples equipped with a berkovich shape indenter. All the samples were carefully ground before analyzing to get the accurate results. For the determination of the E, the Oliver and Pharr method was used.

The tensile strength measurements were also carried out by using Equation (4). It should be noted that for the accuracy, extensive calculations were made in repetition for over 15 values of hardness [54].

(4) $$\sigma_{\mathrm{UTS}}=\frac{H_{V}}{3.34}-56$$

where

$\;\sigma_{\mathrm{UTS}}$ = Tensile strength in MPa
$\;H_{V}$= Hardness in MPa.

## 3. Results

### 3.1. Particle Sizes

**Figure 4:**
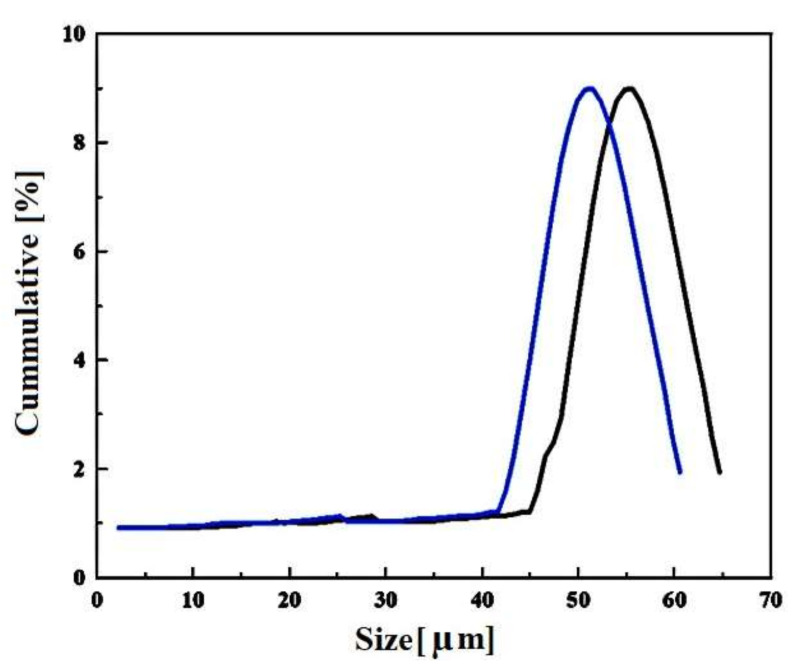
The particle sizes of titanium and tin powders.

Figure 4 depict the particle sizes of the titanium and tin powders. The average size of powders of Ti and Sn are in the range of 50 to 58 µm, respectively. This shows the difference in particle sizes which is beneficial for densification.

### 3.2. Relative Denisty

**Figure 5:**
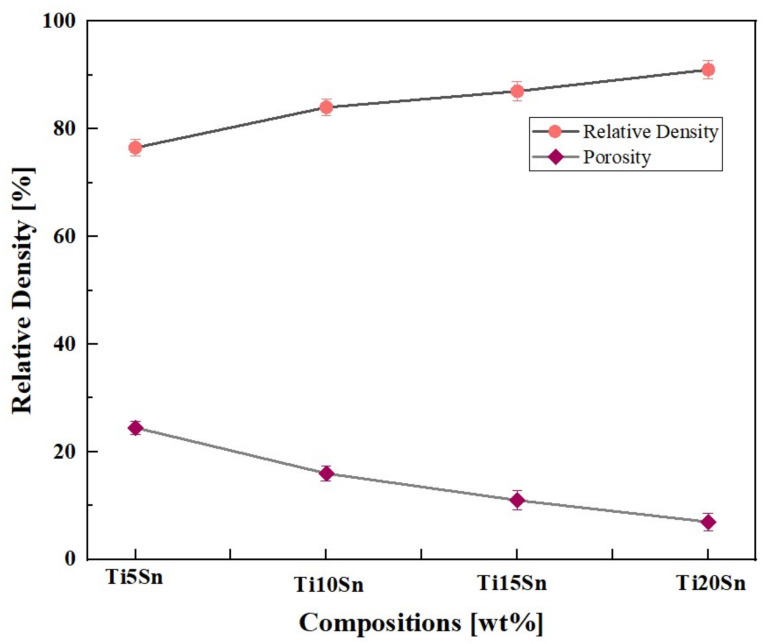
Relative density and porosity of the Ti-Sn alloys.

Figure 5 shows the relative density of the Ti-Sn alloys. The relative densities were increased upon the incremental addition of tin contents. Hence significant reduction in porosity was observed. This infers very good densification.

### 3.3. Microstructure

**Figure 6:**
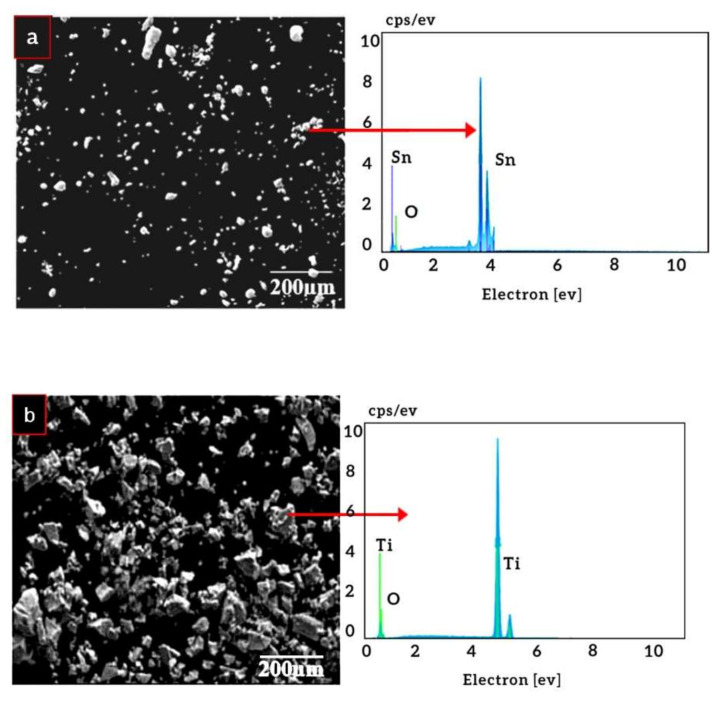
SEM images showing morphology (**a**) tin and (**b**) titanium powders.

Figure 6 shows the morphologies of titanium and tin powders. The titanium powder is irregular in shape which helps in the compaction process since such particles will enhance the compressibility and ultimately densification.

**Figure 7:**
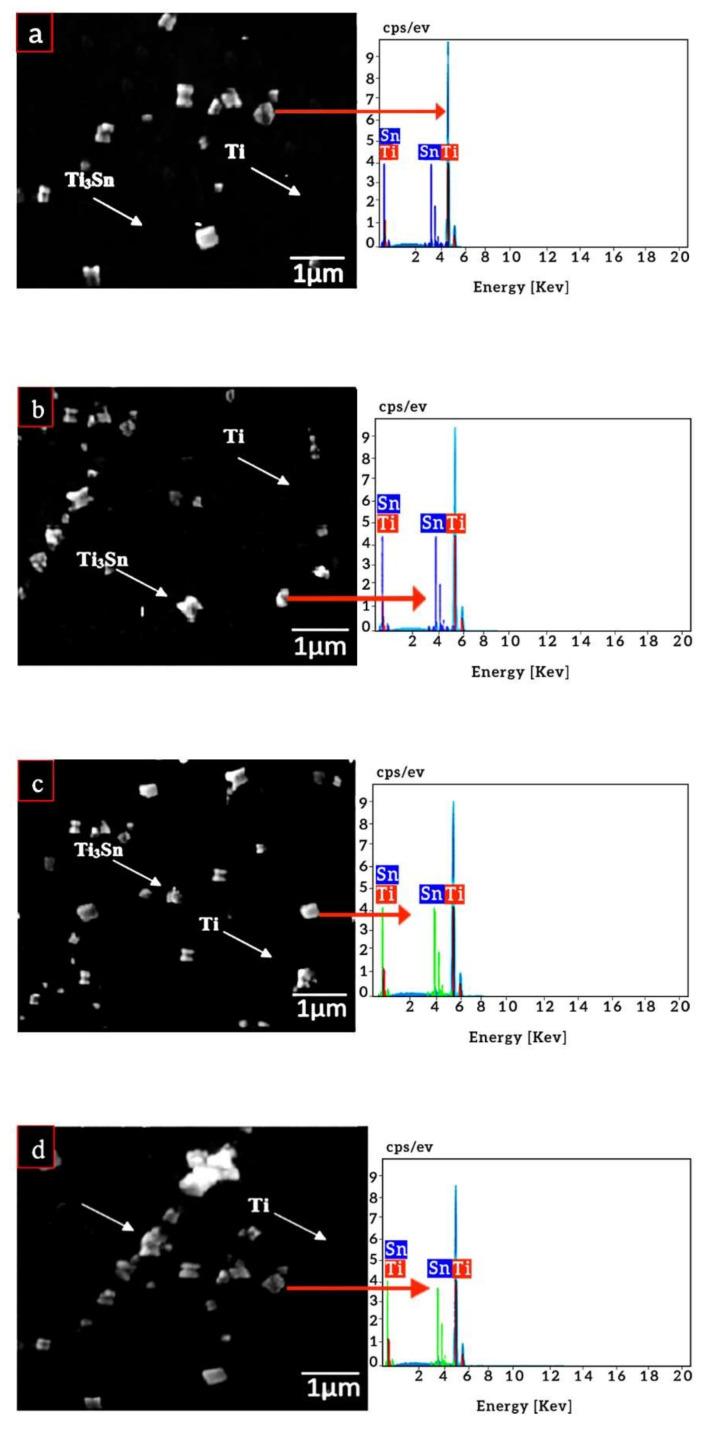
The SEM micrograph of Ti-Sn sintered alloys; (**a**) Ti-5Sn (**b**) Ti-10Sn (**c**) Ti-15Sn and (**d**) Ti-20Sn.

Figure 7 shows the microstructure of the Ti-Sn alloys containing varying percentages of Sn (5%, 10%, 15% and 20%). The alloys exhibited single alpha phase thereby agreeing with the binary diagram of Ti-Sn alloys system. This shows that the solid-solubility of tin in titanium is around 20 wt. %. However, minor porosity was also noticed in alloys.

### 3.4. Phase Composition Analysis

**Figure 8:**
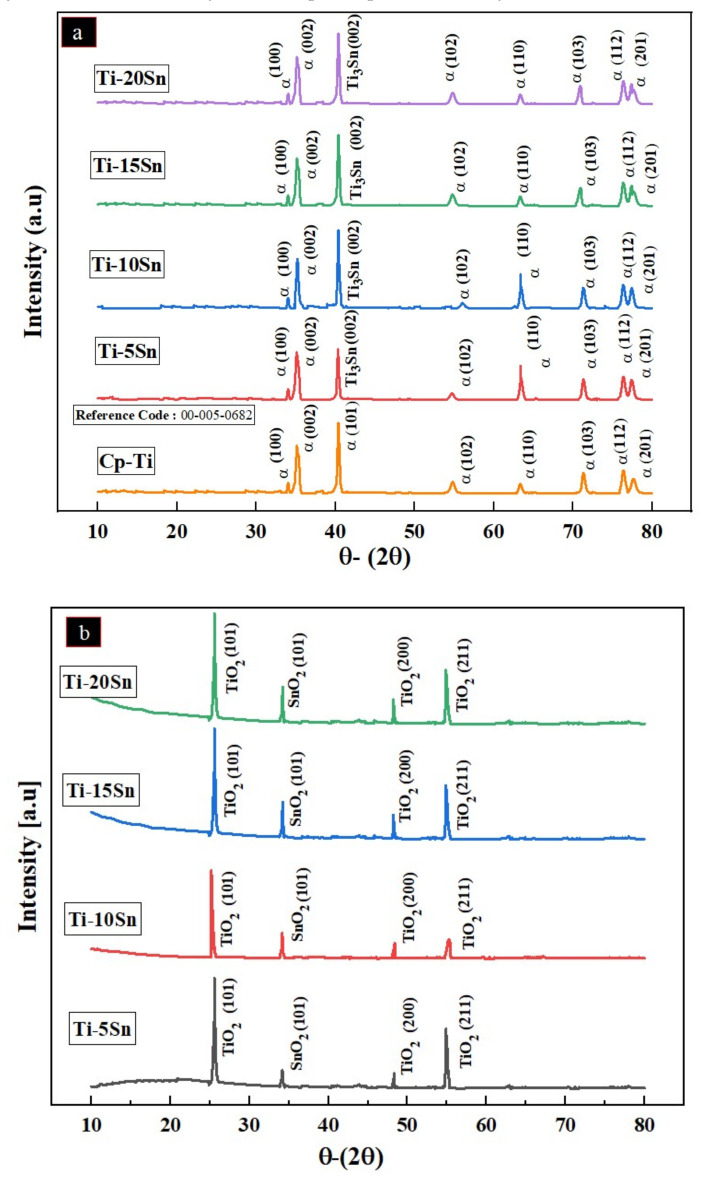
The XRD spectra of alloys and their corrosion products; (**a**) Ti-Sn alloys with reference of the Cp-titanium and (**b**) corrosion products.

XRD of binary Ti–Sn alloys is shown in Figure 8a. The XRD peaks matches the JCPDs file that confirms the existence of titanium, tin, and its compounds (i.e., Ti_3_Sn) [55] in the alloys formed in this study. No beta phase peak was analyzed [56].

Likewise the corrosion products are shown in Figure 8b. Results suggest the formation of TiO_2_ layer on the surface which could be found elsewhere too [57]. Moreover, the SnO_2_ peaks was also formed at the angle of 34° [58].

### 3.5. Corrosion Resistance

**Figure 9:**
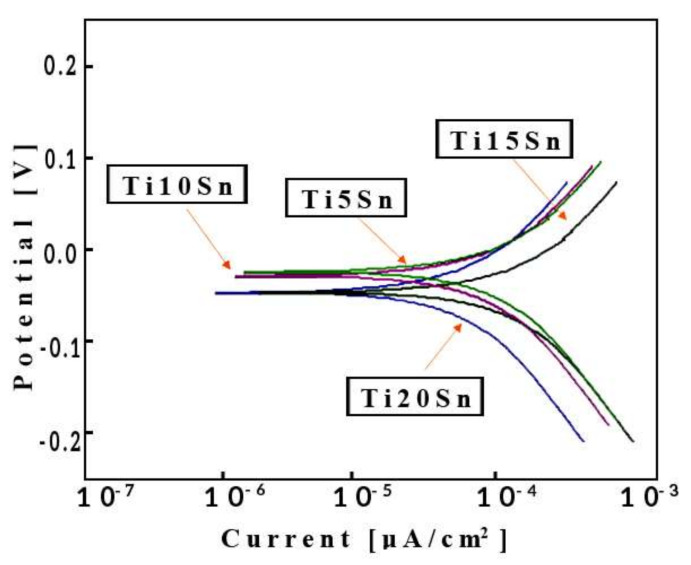
Potentiodynamic polarization curves obtained from the corrosion testing of as-fabricated Ti-Sn alloys in SBF at 37 °C.

Figure 9 depicts the Tafel curve of Ti-Sn alloys. The corrosion rate is decreasing when tin content was increased in SBF solution. The values of I_corr_ and corrosion potential (E_corr_) is directly deduced from the Tafel curve and placed in Table 4.

### 3.6. Mechanical Properties

**Figure 10:**
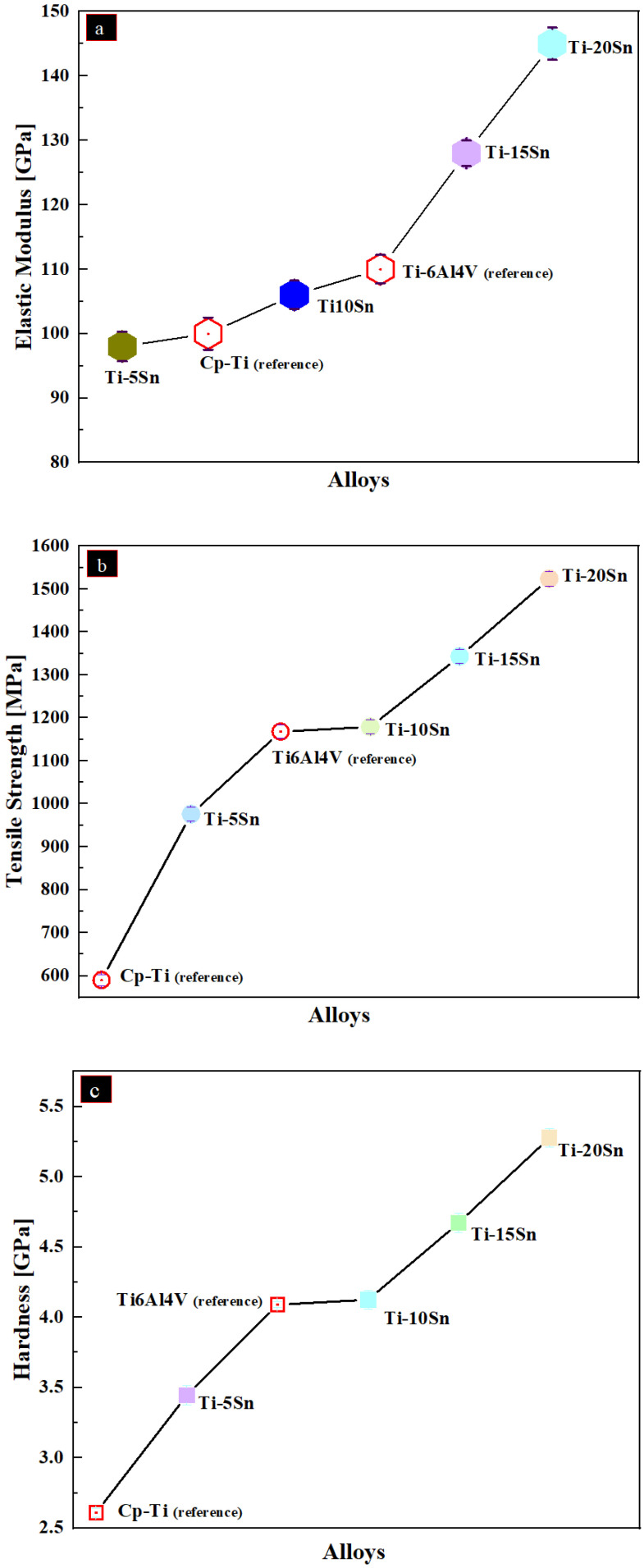
The elastic modulus, tensile strength and hardness of Sintered Ti-Sn alloys; (**a**) Elastic Modulus, (**b**) Tensile Strength and (**c**) Hardness. In addition, the reference values of some alloys are shown.

Figure 10 shows the modulus of elasticity, tensile strength and hardness of Ti-Sn alloys. The linear trend of elastic moduli as noticed, upon incremental Sn content into titanium. Moreover, the hardness of Cp-Ti and Ti6Al4V is reported in literature is 2.61 GPa and 4.09 GPa, respectively [59]. The corresponding calculated elastic moduli is 590 MPa and 1168 MPa, respectively.

## 4. Discussion

### 4.1. Particle Sizes

The varying particle sizes were observed, i.e., 50 to 58 µm respectively. The difference in the particle sizes led to better particle bonding with respect to each other and thus improved sintering of the alloy was observed [43]. This suggests improved densification, i.e., removal of porosity. In addition, the particle sizes were irregular which also facilitates the strong sintering associated densification too.

### 4.2. Relative Density

The relative densities of the samples are shown in Figure 5 which suggests that as Sn content is increased, the relative density is improved. Moreover, addition of tin significantly reduces the densification temperature. This led to enhanced properties and ultimately reduction in porosity was observed [60].

### 4.3. Microstructure

The Ti-Sn alloys containing varying percentages of Sn (5, 10, 15, and 20 wt. %) were prepared and their microstructures were shown earlier in Figure 5. Based on the microstructures, there are two observations: (1) porosity in compacts and (2) formation of intermetallic compounds. The porosity in compacted alloys is due to the slowly cooled alpha titanium [61]. These microstructures contain intermetallic particles/precipitates in the titanium matrix, i.e., Ti_3_Sn. The presence of these particles is due to the supersaturation of the Sn in the titanium matrix. As the tin content increased the precipitation of the particle increased accordingly. This supersaturation leads to reduction in Sn solid solubility in the alpha titanium thereby favoring the formation of Ti_3_Sn [43]. Such intermetallic was observed significantly when Sn quantity increased beyond 10 wt. %. The presence of such particles is beneficial in terms of enhancing the hardness. However, elastic moduli were not enhanced when Sn content was increased from 5 to 10 wt. % than that of reference counterpart (Ti6Al4V). On the contrary, it went up when Sn was incorporated between 15 and 20 wt. %.

### 4.4. Phase Analysis

Figure 7a depicts the phase analysis of the Ti-Sn alloys wherein presence of single alpha phase was observed. The formation of the alpha phase which is characteristic structure was unchanged. Since, presence of Sn in Ti did not affect the XRD peaks owing to very small difference in their atomic radii [62]. Conversely, the Sato et al. measured the transformation range that clearly shows the addition of tin, and also, there was no change observed in the phases of Ti-Sn binary alloys. Therefore, these results are in agreement to what is being reported elsewhere [56]. 

Likewise Figure 7b shows the phase analysis of the corrosion products on the surface of the Ti-Sn alloys. The TiO_2_ and SnO_2_ layers were observed as the corrosion products. The TiO_2_ acts as the superior antibacterial layer [57]. While SnO_2_ is also beneficial in terms of corrosion in human body.

### 4.5. Corrosion Resistance

The utmost property of implant material for long term clinical applications is its ability to resist corrosion in specific environment [63]. This is because, after implantation in living body, those materials which possess low-slung corrosion resistance releases metallic ions in surrounding tissues due to instability of their oxide film(s) thereby causing cytotoxicity. The composition of the implant material has serious influence on the strength of the oxide layer [64]. Figure 9 shows the different variation in current density (I_corr_) for examined alloys as a result of their exposition in the SBF fluid. The values of I_corr_ and corrosion potential (E_corr_) were calculated, as seen in Table 4. It is clear from the value of I_corr_ which is in decreasing order, i.e., from 0.06 to 0.022 µA, that the percentage of the Sn in titanium is increased, whereas Ecorr shows increasing order, i.e., from −201 to −150 mV. This is because when the tin content increases the corrosion rate of the alloy also decreases due to the formation of stable oxide layer (i.e., TiO_2_ and SnO_2_). This layer inhibits the rate of reaction with the body fluid as shown earlier in Figure 9 [65,66]. If the material having a low I_corr_ and high E_corr_ then the material is said to be highly corrosion resistive material [67]. Comparatively, the I_corr_ value of Ti-Sn and its alloys is lower than that of the standard Cp- Ti and Ti6Al4V alloys. Since the Icorr value of Cp-Ti and Ti6Al4V is 0.70 µm/cm^2^ and 1.50 µm/cm^2^ respectively. These values are higher than that of Ti10Sn, Ti15Sn, and Ti20Sn alloys [68,69]. This shows their excellent corrosion resistance.

### 4.6. Mechanical Properties

The addition of tin in titanium matrix has been studied elsewhere. For example, incorporation of Sn the drastic increase in the intermetallic compounds which ultimately leads to the enhancement in the elastic modulus. This has been shown in earlier Figure 8. The elastic modulus of the Ti-5Sn and Ti-10Sn is comparable to that of Ti6Al4V counterpart, i.e., 112 GPa [70]. However, upon incremental concentration above 10% of tin leads to increased elastic modulus as witnessed in Ti-15% Sn and Ti-20%Sn alloys. This is attributed to the formation of Ti_3_Sn HCP alpha phase which could be found elsewhere too [71].

Moreover, Lai-Chang et al. reported that the hardness value of Ti6Al4V to be 4.09 GPa and the calculated tensile strength to about 1168 MPa. These values are comparable to the Ti10Sn alloy produced in present study [72]. Additionally, the hardness of Ti-10Sn is also comparable to the reference alloy, i.e., Ti6Al4V. In contrast, the other alloys, i.e., beyond 10 wt. % Sn exhibited higher hardness while Ti-5Sn showed lower values. Thus, properties of Ti-10Sn alloy is comparable Ti6Al4V and can be beneficial for biomaterial applications upon further studies.

## 5. Conclusions

Titanium-based alloys with varying amounts of Sn (5 to 20 wt. %) were developed via powder metallurgy to better understand their behavior for potential applications such as biomedical implants. Corrosion and mechanical properties were examined. Based on the present set of experimental conditions, the following are concluding remarks.

The alloy was found to contain alpha phase at all concentrations of Sn. At 10. wt. % of Sn, the elastic modulus was comparable to that of reference alloy, i.e., Ti6Al4V. Similarly, hardness and tensile strengths were also increased in general, and specifically, at 10 wt. % Sn, the values were comparable to that of the reference alloy. The corrosion rate of Ti-Sn alloys (as of this study) and reference alloy (Ti6Al4V) were also compared. The corrosion trend was in decreasing order upon the addition of Sn. The corrosion rate at 20 wt. % Sn was found to be minimum among Ti-Sn alloys including reference counterpart. Therefore, addition of Sn was found to be promising for future applications of titanium-based alloys.

## Figures and Tables

**Table 1:** Shows the materials used in this study.

S.No.	Materials	Purity [%]
1.	Titanium powder	99.99
2.	Tin powder	99.98

**Table 2:** The alloys produced in this study via powder metallurgy.

S.No.	Sn (wt. %)	Ti (wt. %)	Alloys
1.	5.0	95.0	Ti-5Sn
2.	10.0	90.0	Ti-10Sn
3.	15.0	85.0	Ti-15Sn
4.	20.0	80.0	Ti-20Sn

**Table 3:** The chemical composition of SBF solution.

S.No.	Chemicals	Amount
1.	NaCl	6.559 g
2.	Na_2_CO_3_	2.26 g
3.	KCl	0.3773 g
4.	K_2_HPO_4_	0.1496 g
5.	H_12_Cl_2_MgO_6_	0.3411 g
6.	HCL	10 mL
7.	CaCl_2_	0.3635 g
8.	Na_2_SO_4_	0.0731 g
9.	Tris	6.0662 g
10.	Distilled H_2_O	960 mL

**Table 4:** Corrosion test results by using Tafel extrapolation.

Composition	Ecorr (mv)	Icorr (µA)	Corrosion Rate (mm/Year)
Ti-5Sn	−201	1.50	1.688 × 10^−8^
Ti-10Sn	−194	0.54	5.89 × 10^−9^
Ti-15Sn	−171	0.43	4.58× 10^−9^
Ti-20Sn	−150	0.37	3.65 × 10^−9^

## Data Availability

Data sharing not applicable.
